# Trolox and recombinant Irisin as a potential strategy to prevent neuronal damage induced by random positioning machine exposure in differentiated HT22 cells

**DOI:** 10.1371/journal.pone.0300888

**Published:** 2024-03-21

**Authors:** Roberto Bonanni, Ida Cariati, Anna Maria Rinaldi, Mario Marini, Giovanna D’Arcangelo, Umberto Tarantino, Virginia Tancredi

**Affiliations:** 1 Department of Biomedicine and Prevention, “Tor Vergata” University of Rome, Rome, Italy; 2 Department of Systems Medicine, “Tor Vergata” University of Rome, Rome, Italy; 3 Centre of Space Bio-Medicine, “Tor Vergata” University of Rome, Rome, Italy; 4 Department of Clinical Sciences and Translational Medicine, “Tor Vergata” University of Rome, Rome, Italy; Shaanxi Provincial People’s Hospital, CHINA

## Abstract

Neuronal death could be responsible for the cognitive impairments found in astronauts exposed to spaceflight, highlighting the need to identify potential countermeasures to ensure neuronal health in microgravity conditions. Therefore, differentiated HT22 cells were exposed to simulated microgravity by random positioning machine (RPM) for 48 h, treating them with a single administration of Trolox, recombinant irisin (r-Irisin) or both. Particularly, we investigated cell viability by MTS assay, Trypan Blue staining and western blotting analysis for Akt and B-cell lymphoma 2 (Bcl-2), the intracellular increase of reactive oxygen species (ROS) by fluorescent probe and NADPH oxidase 4 (NOX4) expression, as well as the expression of brain-derived neurotrophic factor (BDNF), a major neurotrophin responsible for neurogenesis and synaptic plasticity. Although both Trolox and r-Irisin manifested a protective effect on neuronal health, the combined treatment produced the best results, with significant improvement in all parameters examined. In conclusion, further studies are needed to evaluate the potential of such combination treatment in counteracting weightlessness-induced neuronal death, as well as to identify other potential strategies to safeguard the health of astronauts exposed to spaceflight.

## Introduction

The presence of gravitational forces is a fundamental condition for the proper development and maintenance of homeostasis of many physiological systems [1,2]. In fact, in the absence of gravity, numerous structural and functional alterations occur in the various organs and systems of the body [3]. The most well-known and obvious effects of weightlessness are the reduction of bone mineral density (BMD) and loss of muscle mass and strength, conditions typical of the major musculoskeletal disorders associated with aging, such as osteoporosis and sarcopenia [4–6]. In addition, the elimination of the normal hydrostatic pressure gradient induced by weightlessness leads to cardiovascular dysfunction, depression of the immune response and metabolic alterations [7–9].

Recently, alterations have also been reported in the central nervous system (CNS) related to the function or expression of important biomarkers, such as receptors, ion channels, neurotrophins, and neurotransmitters, resulting in impaired neuronal homeostasis that could negatively impact cognitive function and learning and memory processes [10]. In this regard, Popova et al. reported that mice exposed to one month of spaceflight on the Russian biosatellite BION-M1 showed significant alterations in the expression pattern of genes involved in dopamine synthesis and degradation, while reduced expression of the serotonergic 5-HT2A receptor was detected at the hypothalamic level [11]. In addition, reduced expression of the gene coding for glial cell-derived neurotrophic factor (GDNF) in the striatum and hippocampus and the gene coding for brain dopaminergic neurotrophic factor (CDNF) in the substantia nigra was observed, suggesting deregulation in the control of GDNF and CDNF genes among the main responsible of the deleterious effects of spaceflight on the dopaminergic system [12].

Changes in brain structure and function have also been reported in astronauts exposed to long-duration spaceflight, including sleep and visual disturbances, reduced attention and executive functions, cognitive deficits, and alterations in brain morphometry, all of which are associated with an increased rate of neuronal apoptosis [13]. Not surprisingly, B-cell lymphoma 2 (Bcl-2) expression, an anti-apoptotic marker, was found to be reduced in the striatum and hypothalamus of mice exposed to spaceflight [14]. In addition, Wise and colleagues found increased levels of reactive oxygen species (ROS) and the absence of reduced glutathione (GSH) in the brainstem and frontal cortex of mice subjected to hindlimb unloading, suggesting the apoptotic death process initiation underlying these events [15]. Similarly, Pani and colleagues evaluated the impact on cell viability by an Annessin V assay on primary murine cerebral cortex neurons exposed to random positioning machine (RPM) for short (1 h), medium (24 h) and long-term (10 days) [16]. The treatment resulted in a significant increase in apoptotic neurons and a marked alteration in neuronal morphology, in terms of neurite length and soma size. The authors also reported that the area and length of the neuritic network were affected by RPM exposure, as evidenced by changes in the cellular cytoskeleton organization [16]. In agreement, Sun et al. observed by TUNEL assays the apoptotic cells presence in the cortex and hippocampus of rats subjected to simulated weightlessness, correlating this process of neuronal death with the impairment of cognitive functions of learning and memory [17].

Interestingly, a close association between the space environment exposure and alterations in the expression of proteins involved in neuronal structure and metabolic function was also recently observed by Mao et al. who characterized proteomic changes in the brains of mice upon return from a 13-day Space Shuttle Atlantis mission. Proteomic analysis showed alteration of 26 proteins involved in synaptic plasticity in gray and white matter after spaceflight, confirming the impact of the absence of load on brain structure and integrity [18]. Finally, Chen and colleagues investigated the effects of simulated microgravity for 7 and 21 days on the hippocampus, cerebral cortex, and striatum in a tail suspension rat model, finding reduced expression of brain-derived neurotrophic factor (BDNF), marked oxidative stress and atrophy of neurons in the cerebral cortex after 21 days of exposure. Interestingly, a gradual suppression of inflammatory cytokines was detected in the striatum, suggesting that the simulated microgravity impact on the brain is closely dependent on the exposure time and brain regions involved [19].

Despite this evidence, the mechanisms underlying the effects of simulated microgravity on the CNS have yet to be extensively elucidated, just as potential strategies to prevent cognitive decline related to no load are unknown. In this context, recombinant Irisin (r-Irisin) could provide a valuable support in the neuroprotection of astronauts, as this hormone is known to promote BDNF up-regulation and neurogenesis, ameliorating depressive behaviour, apoptosis and oxidative damage in various pathological conditions [20–22]. Furthermore, r-Irisin has been shown to be effective in preventing musculoskeletal damage induced by simulated microgravity in several experimental studies, highlighting its utility in the problem of neuronal death induced by weightlessness [23]. However, Tsiani et al. reported that the half-life of this potent substance may be limited to a few hours, suggesting its limited efficacy in medium- and long-term treatments [24]. Therefore, additional tools are needed to ensure the neuronal health of space crews exposed to microgravity for long periods. In this context, 2,5,7,8-tetramethylchroman-2-carboxylic acid (Trolox), a known water-soluble analogue of vitamin E, could represent a valid protective strategy for neurons exposed to RPM. Indeed, vitamin E has been shown to enhance BDNF expression, reduce oxidative stress and prevent neuronal death under different experimental conditions [25–28]. In this regard, antioxidant strategies have shown promise in preserving cellular health under microgravity conditions, highlighting the crucial role of oxidative stress in cell death and suggesting the need for antioxidant strategies as prevention [29].

Therefore, the aim of our work was to test the efficacy of a single administration of Trolox, r-Irisin or a combined treatment in counteracting neuronal death and cognitive impairment associated with this condition. Specifically, we exposed differentiated HT22 cells to RPM for 48 h and analyzed the effects of these three different treatments on the expression of markers associated with cell viability and cognitive function, proposing the combined administration of Trolox and r-Irisin as a potential therapeutic strategy to prevent and/or counteract the cell damage that characterizes astronauts exposed to spaceflight.

## Materials and methods

### Cell cultures

HT22 cells were developed from their analogous HT4 cells, immortalized from primary mouse hippocampal neurons. Cells were maintained at 37°C, 10%, CO_2_ in Dulbecco’s Modified Eagle’s Medium (DMEM, Sigma Aldrich–D6546) supplemented with 10% heat-inactivated Fetal Bovine Serum (FBS) and kept at less than 50% of confluence. HT22 cells under proliferation conditions do not express cholinergic and glutamatergic receptors. However, in a selective medium, these cells can undergo a process of differentiation, changing morphology and inducing the expression of specific markers of mature neurons [30,31].

Differentiation was carried out in Neuro-Basal Medium (ThermoFisher, Waltham, MA, USA, 21103–49) containing N2 supplement (Gibco-17502048), at least for 24 h before use. In this condition, the expression of choline acetyltransferase (ChAT), vesicular acetylcholine transporter (VAChT), high-affinity choline transporter (HACT), muscarinic M1 and M2 subunits of Ach- receptors, as well as N-methyl-D-aspartate receptor (NMDARs) and monosialic ganglioside 1 (GM1) was observed [32,33].

### Immunofluorescence

An immunofluorescence analysis was conducted to verify successful differentiation in HT22 cells by investigating the expression of proteins typical of mature neurons, such as N-methyl-D-aspartate receptor 1 (NMDAR1), microtubule associated protein 2 (MAP2) and ChAT. In addition, the same analysis was conducted to investigate the expression of NADPH oxidase 4 (NOX4), an indicator of oxidative stress. In detail, after fixation in 4% paraformaldehyde dissolved in 0.9% saline solution for 30 min, cell cultures were pretreated with ethylenediaminetetraacetic acid (EDTA) citrate, pH 7.8 for 20 min at 95°C, and incubated for 1 h with mouse monoclonal anti-NMDAR1 antibody (556308, BD Pharmingen™, United Sates), rabbit polyclonal anti-MAP2 antibody (ab32454, AbCam, Cambridge, United Kingdom), mouse monoclonal anti-ChAT antibody (CL3173, Novus Biologicals, Littleton, CO, United Sates), or rabbit polyclonal anti-NOX4 antibody (NB110-58849, Novus Biologicals, Littleton, CO, United Sates). Reaction was revealed by using secondary antibodies (715-545-150, 711-545-152, Alexa Fluor® 488, Jackson ImmunoResearch). Washing was performed with PBS/Tween20 pH 7.6 (UCS Diagnostic, Rome, Italy). Finally, cells were counteracted with 4′,6-diamidino-2-phenylindole (DAPI) counterstain (Kreatech Biotechnology B.V., Amsterdam, Netherlands). Images were visualized by a Nikon upright microscope ECLIPSE Ci-S (Nikon Corporation, Tokyo, Japan) connected to a Nikon digital camera and acquired at 40× magnification using NIS-Elements software (5.30.01; Laboratory Imaging, Prague, Czech Republic).

### Simulation experiment by RPM

The RPM system (Airbus Defence and Space Netherlands B.V.) was used to simulate the biological effects of microgravity on differentiated HT22 cells [34]. All experiments were carefully planned according to procedures previously described [35,36]. The rotating RPM frame was placed inside a cell culture CO_2_ incubator. The software responsible for controlling the motion of RPM employed a tailored algorithm, which rotated with a random speed in such a way that the mean gravity vector reliably converged to zero over time, and it concurrently reduced fluid motion in the culture flask. The samples were positioned compactly in the center of rotation, to avoid artifacts and to minimize centrifugal acceleration. All cell samples were carefully processed for in vitro cultivation. We used 24-well plates sealed with dialysis membrane (Visking Medicell International Ltd, Liverpool Road-London code DTV12000.06.000 MWCO 12/14 KDa). The dialysis membrane was deposited on the convex liquid meniscus of the medium inside the well, allowing it to be sealed and thus preventing the formation of air bubbles. The nitrocellulose discs were fixed to the support by means of a rubber ring.

Differentiated HT22 cells were exposed to simulated microgravity regime for 48 h; while plates exposed to normogravity regime were kept in incubator for the same period, so that all cell samples shared the same experimental conditions.

### Differentiated HT22 cells conditioned with Trolox, r-Irisin or combined treatment

The role of Trolox and Irisin in preventing and/or counteracting cell damage induced by RPM exposure was studied by treating cell cultures with Trolox, r-Irisin or both. Specifically, cells were seeded in a 24-well plate at a density of 4 × 10^4^ cells/well. Cell cultures were incubated with 1 × 10^−4^ M Trolox (S-238815, Sigma Aldrich, St. Louis, MO, USA), 10 ng/mL r-Irisin (AG-20B-0153, AdipoGen® Life Sciences, Liestal, Switzerland) or combined treatment, and exposed to RPM for 48 h. Brightfield images of differentiated HT22 cells before and after exposure to RPM were acquired at 40x magnification using NIS-Elements software (5.30.01; Laboratory Imaging, Prague, Czech Republic) and shown in S1 Fig for each experimental condition. Subsequently, treated cell cultures were subjected to the same experimental procedures as untreated samples.

### Cell viability evaluation

A CellTiter 96 AQueous One (Promega, Madison, WI, United States) is a colorimetric method used to identify viable cells [37]. The CellTiter 96 AQueous Assay consists of a novel tetrazolium compound (3-(4,5-dimethylthiazol-2-yl)-5-(3-carboxymethoxyphenyl)-2-(4-sulfophenyl)-2H-tetrazolium-MTS) and an electron-coupling reagent (phenazinemethosulfat-PMS). MTS is bioreduced by cells into a formazan product that is soluble in tissue culture medium. The absorbance of formazan at 490 nm can be measured by using a microplate reader (Spark Multimode Microplate Reader—Tecan, Austria) and it is directly proportional to the number of living cells in the culture. Briefly, using 96-well plates, 20 μL of MTS/PMS solution was added to 100 μL of Hank’s balanced salt solution (HBSS) in each well and incubated for at least 2 h at 37°C. The final concentrations of MTS and PMS were 333 μg/mL and 25 μM, respectively. In the case of 24-well plates, the volumes of MTS/PMS solution and HBSS were doubled to keep the respective concentrations constant. For each condition, the experiment was conducted in triplicate (*n* = 15 from *N* = 5 experiments). All data obtained from the MTS assay are shown in S1 Table.

Trypan Blue staining (Trypan Blue solution, 0.4%, ThermoFisher Scientific, Grand Island, New York) was used to evaluate the presence of dead cells. After RPM exposure, the cell solution was mixed 1:1 with Trypan Blue staining and then fixed with 4% paraformaldehyde for 15 min. Images were acquired at 20x magnification using NIS-Elements software (5.30.01; Laboratory Imaging, Prague, Czech Republic).

### Western blotting analysis

Western blotting analysis was conducted to investigate the expression levels of Akt, Bcl-2 and BDNF in differentiated HT22 cells. Cell proteins extracted by using RIPA buffer were separated by 8%–16% precast SDS-PAGE (Bio-Rad, Hercules, CA, United States) under reduced conditions. Protein concentration was determined using the Pierce BCA Protein Assay Kit (Thermo Scientific, Vilnius, Lithuania). Equal amounts of protein (25 μg) were resolved on 8%–16% precast SDS-PAGE and transferred to PVDF membrane. Then membranes were incubated with rabbit monoclonal anti-Akt (#4685 Cell Signalling Technology), mouse monoclonal anti-Bcl-2 (#15071 Cell Signalling Technology), or mouse monoclonal anti-BDNF [3B2] (clone ab205067, AbCam, Cambridge, United Kingdom) and successively with anti-rabbit IgG coupled to HRP or anti-mouse IgG coupled to HRP, respectively. Moreover, the same membranes were incubated with mouse monoclonal anti-GAPDH (ab8245, AbCam) used for normalization. Immunoreactive electrophoretic bands were detected by enhanced chemiluminescence (ECL Advance, Amersham; GE Healthcare Life Sciences, Little Chalfont, Buckinghamshire, United Kingdom) using a VersaDoc 5,000 Imager (Bio-Rad). The expression levels of Akt, Bcl-2 and BDNF under the different experimental conditions were quantified by calculating the densitometric values of the relevant bands and normalizing the results against the GAPDH expression, expressing them as mean ± standard deviation. The original western blotting images are shown in S2B–S2E Fig. All data obtained from western blotting analysis for Akt, Bcl-2 and BDNF are shown in S2, S3 and S6 Tables, respectively.

### Measurement of intracellular ROS level

The fluorescent probe 2’,7’-dichlorodihydrofluorescein di-acetate (H2DCFDA) (D399, InvitrogenTM, ThermoFisher Scientific, USA) was used to detect any changes in the level of intracellular ROS induced by RPM exposure in differentiated HT22 cells. In detail, all cell samples were washed several times with PBS and incubated with 10 μM H_2_DCFDA for 40 min at 37°C in the dark after RPM exposure. A plate reader (Spark Multimode Microplate Reader-Tecan, Austria) was used to measure the mean fluorescence intensity of each experimental group, representing the intracellular ROS level [38]. For each condition, the experiment was conducted in triplicate (*n* = 15 from *N* = 5 experiments). All data obtained from measurement of intracellular ROS level are shown in S4 Table.

### Immunocytochemistry

BDNF expression was also investigated in differentiated HT22 cells by immunocytochemical analysis. After fixation in 4% paraformaldehyde for 15 min, the cell samples were pre-treated with EDTA citrate (pH 7.8) for 30 min at 95°C and then incubated for 1 h with mouse monoclonal anti-BDNF [3B2] (clone ab205067, AbCam, Cambridge, United Kingdom; 1:100). Washings were performed with PBS/Tween20 (pH 7.6) (UCS Diagnostic, Rome, Italy). The immunocytochemical reaction was detected using the horse-radish peroxidase (HRP)-3,3′diaminobenzidine (DAB) detection kit (UCS Diagnostic, Rome, Italy). Specifically, 50 μL of DAB/450 μL of substrate were incubated for 3 min. To assess the immunostaining background, we included negative controls for each reaction by incubating sections with secondary antibodies (HRP) alone or a detection system (DAB) alone (S2A Fig). Immunopositive cells for BDNF were detected using NIS-Elements software (5.30.01; Laboratory Imaging, Prague, Czech Republic) and expressed as a percentage of the total analyzed for BDNF. For each condition, the experiment was conducted in triplicate (*n* = 15 from *N* = 5 experiments). All data obtained from immunocytochemistry are shown in S5 Table.

### Statistical analysis

Statistical analysis was performed using GraphPad Prism 8 Software (Prism 8.0.1, La Jolla, CA, USA). Data were expressed as mean ± standard deviation and were compared by one-way ANOVA and Tukey’s multiple comparison test. For all procedures, data were considered significantly different if *p*<0.05.

## Results

### HT22 cell differentiation promotes the expression of proteins typical of mature neurons

The successful differentiation of HT22 cells was investigated by immunofluorescence analysis, assessing the expression of NMDAR1, MAP2 and ChAT, which are typical proteins of mature neurons.

**Fig 1:**
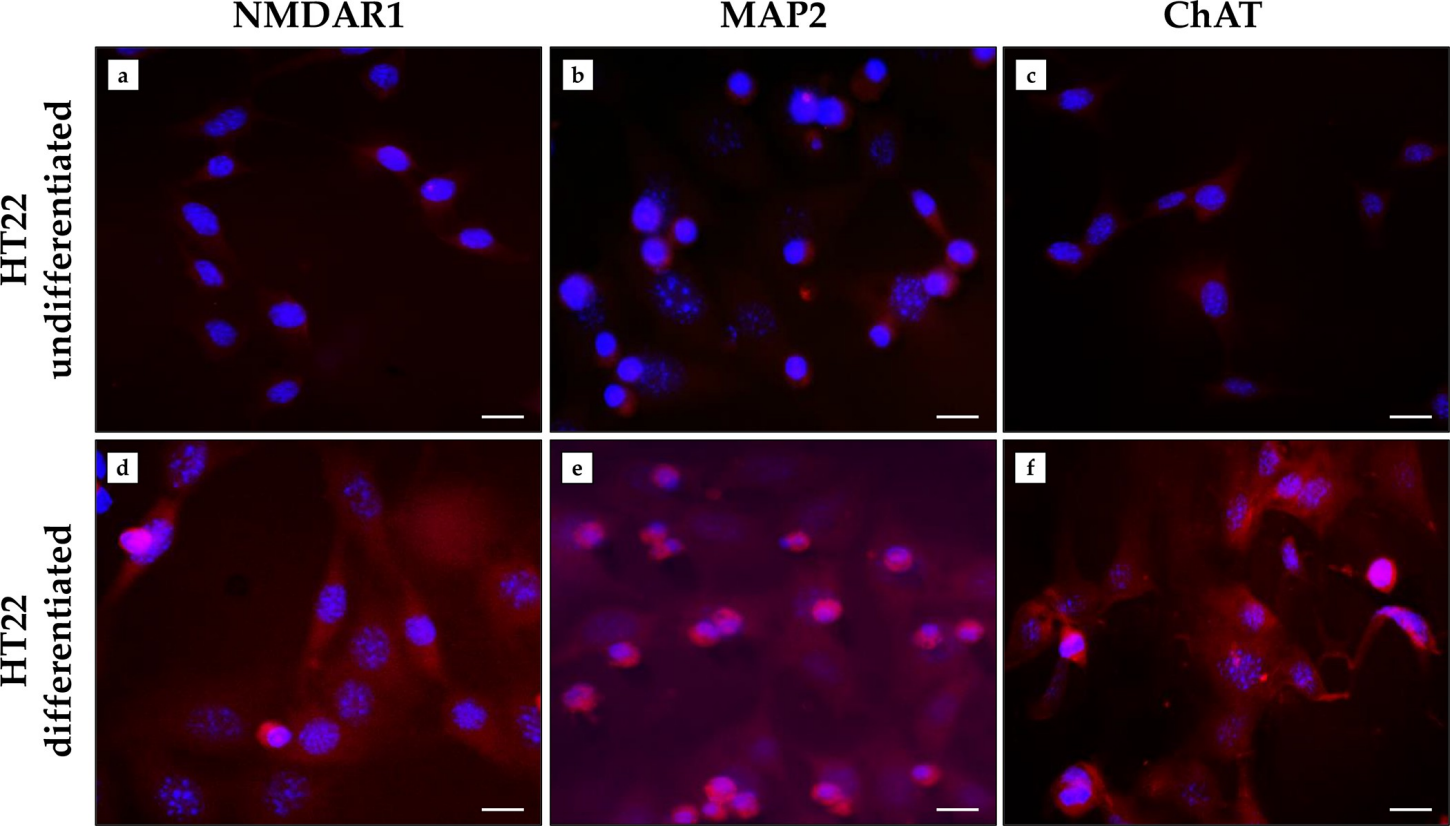
Evaluation of the differentiation process in HT22 cells by immunofluorescence analysis. (a–c) Nuclei are stained with DAPI (blue) in HT22 undifferentiated cells. (d–f) N-methyl-D-aspartate receptor 1 (NMDAR1), microtubule associated protein 2 (MAP2) and choline acetyltransferase (ChAT) immunostaining is depicted in red in HT22 differentiated cells. 40× images, scale bar represents 100 μm.

Fig 1 shows that treatment of undifferentiated HT22 cells with Neurobasal medium containing N2 supplement successfully promoted differentiation. Indeed, a marked fluorescent signal for NMDAR1 (Fig 1D), MAP2 (Fig 1E) and ChAT (Fig 1F) was detected in differentiated HT22 cells, whereas the expression of these proteins was absent or nearly absent in undifferentiated HT22 cells (Fig 1A–1C).

### Efficacy of different treatments on cell viability after RPM exposure

The effects of treatment with Trolox, r-Irisin, or both on cell viability were evaluated in differentiated HT22 cells after RPM exposure by MTS assay, Trypan Blue staining, western blotting analysis, measurement of intracellular ROS levels and immunofluorescence for NOX4.

**Fig 2:**
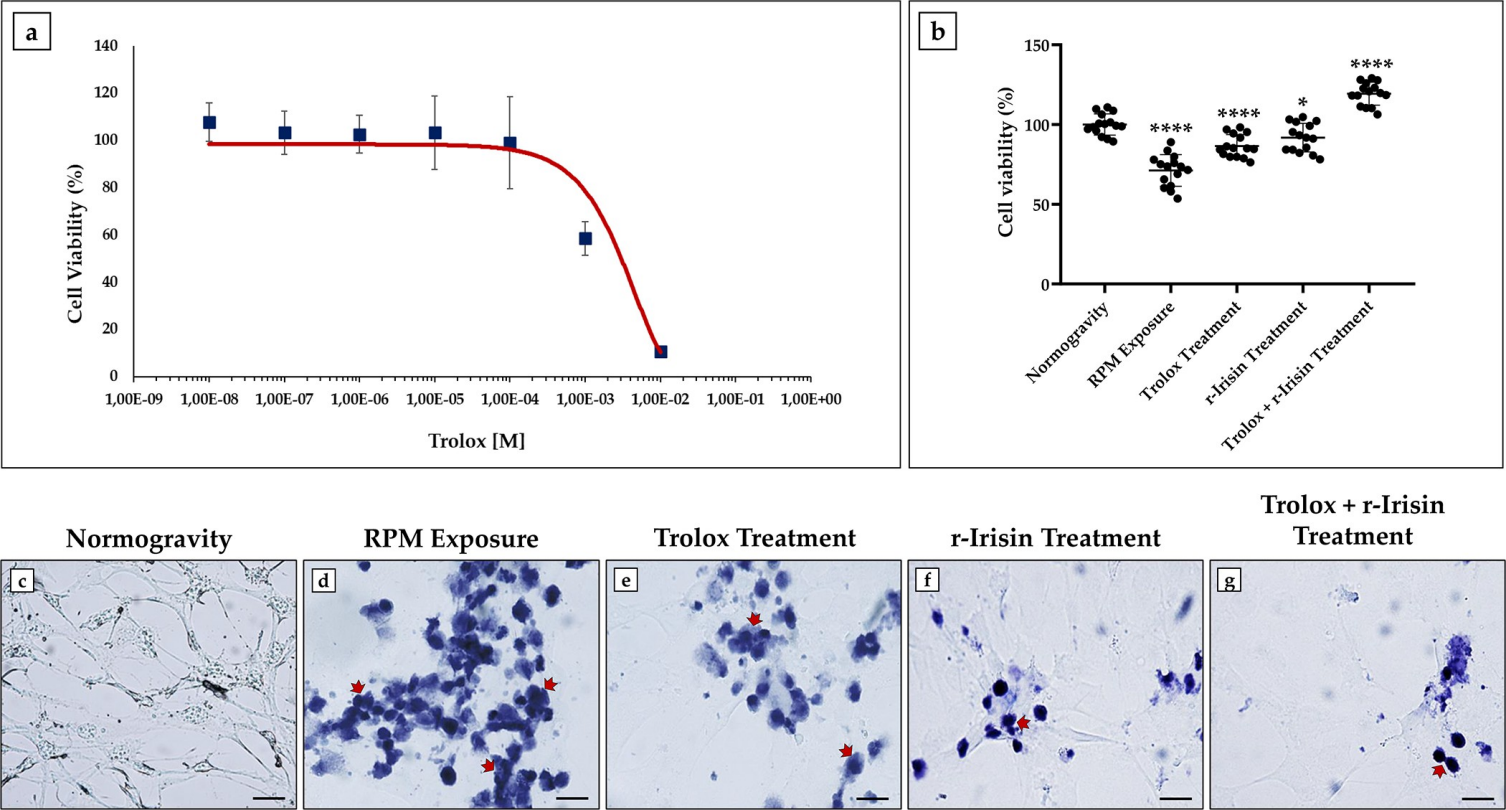
Effects of treatment with Trolox, r-Irisin or both on viability in differentiated HT22 cells. (a) A dose-response curve was constructed to establish the nontoxic dose of Trolox, and cell viability was assessed by MTS assay (*n* = 15 from *N* = 5 experiments). The half inhibitory concentration (IC50) was obtained at a dose of 1 × 10^−3^ M. (b) Cell viability by MTS assay was significantly reduced after RPM exposure compared to the normogravity regime (****p<0.0001). Treatment with Trolox (****p<0.0001) or r-Irisin (*p<0.05) promoted a partial recovery of cell viability, whereas the combined treatment completely preserved the viability of RPM-exposed cells with significantly higher values compared to the normogravity regime (****p<0.0001) (*n* = 15 from *N* = 5 experiments). Data were expressed as mean ± standard deviation and were compared by one-way ANOVA and Tukey’s multiple comparison test. (c–g) Trypan Blue staining: In normogravity (c), HT22 cells were viable and well differentiated, whereas RPM exposure (d) promoted neuronal death and a tendency to form deep blue stained cell clusters (arrows); treatment with Trolox (e) and r-Irisin (f) partially preserved cell viability, while complete recovery was observed after combined treatment (g).

First, a dose-response curve was constructed to estimate the dose of Trolox for which nontoxic effects are detected by treating differentiated HT22 cells with increasing concentrations of the substance and then assessing cell viability by MTS assay. As shown, treatment did not affect cell viability up to a dosage of 1 × 10^−4^ M, whereas a progressive reduction was observed at higher concentrations of Trolox. Importantly, half inhibitory concentration (IC50) was obtained at a dosage of 1 × 10^−3^ M (Fig 2A). Based on these results, differentiated HT22 cells were treated with Trolox, r-Irisin or both and exposed to RPM for 48 h.

Cell viability was investigated by MTS assay and the results are shown in Fig 2B. Not surprisingly, a significant reduction in cell viability was detected in cells exposed to RPM (71.2 ± 9.9; ****p<0.0001) compared to cells in normogravity (100.0 ± 6.6). A partial recovery of cell viability was observed in RPM-exposed cells treated with Trolox (86.6 ± 7.1; ****p<0.0001) or r-Irisin (91.8 ± 8.9; *p<0.05) compared to the normogravity regime. Interestingly, the combined treatment with Trolox and r-Irisin completely counteracted the damage induced by RPM exposure, with significantly elevated cell viability values (119.3 ± 7.1; ****p<0.0001) respect to control cells.

These results were confirmed by Trypan Blue staining, which allowed a qualitative analysis to distinguish viable from dead cells, both under normal conditions and after exposure to RPM (Fig 2C–2G). In fact, Trypan Blue is a dye that can selectively stain dead cells, whereas live cells do not allow it to penetrate the cytoplasm because they have an intact cell membrane. In normogravity conditions, Trypan Blue staining revealed the presence of few dead cells within a well-defined neuronal network formed by viable differentiated HT22 cells (Fig 2C). In contrast, a pronounced increase in the presence of dead cells was detected after RPM exposure, in association with the tendency to form cell clusters, as evidenced by the large areas of cell aggregation where blue staining increased in intensity (Fig 2D). Interestingly, treatment with Trolox or r-Irisin promoted partial recovery of cell viability (Fig 2E and 2F), while the combined treatment of Trolox and r-Irisin was effective in preserving cell viability (Fig 2G).

**Fig 3:**
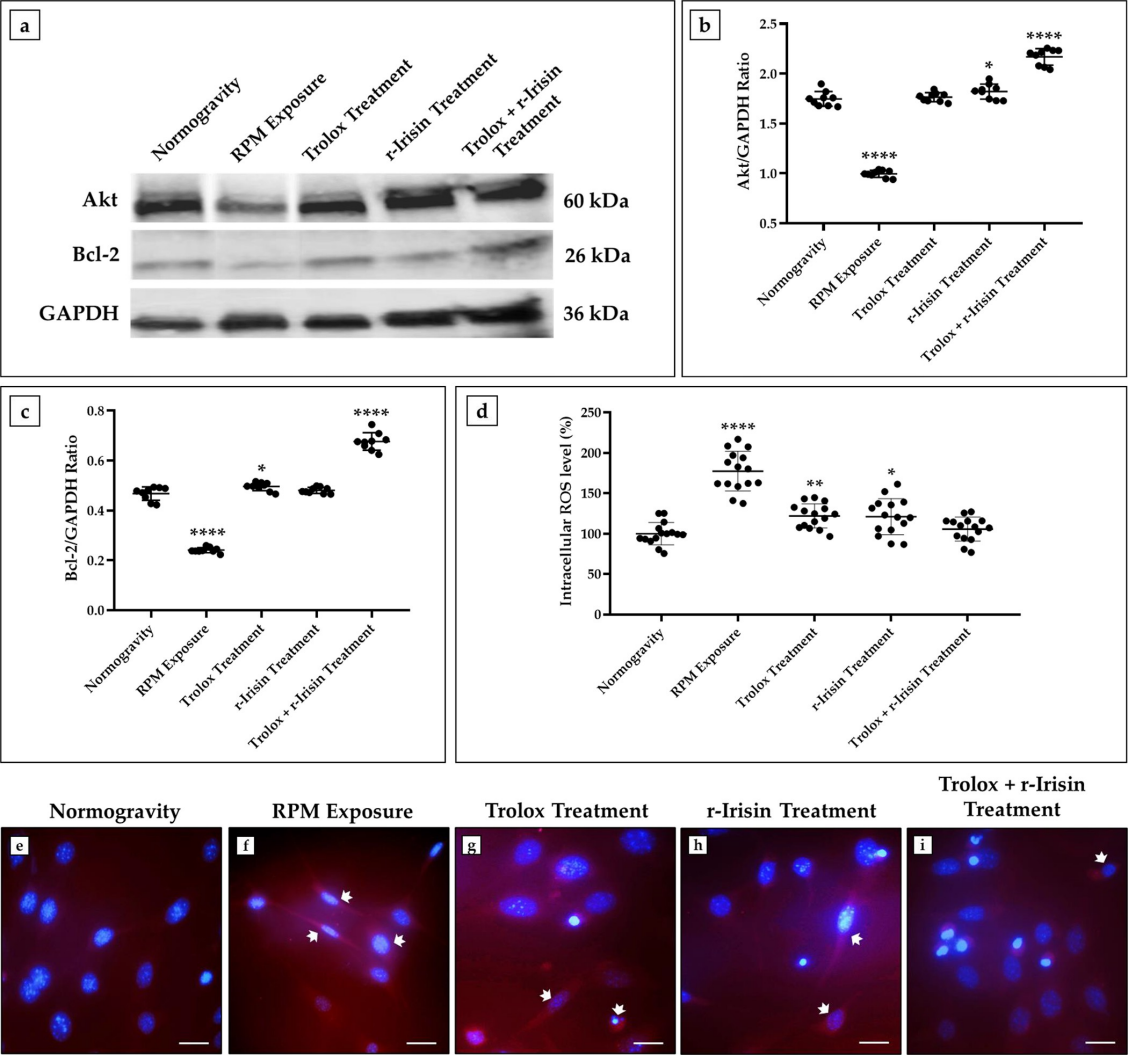
Effects of treatment with Trolox, r-Irisin or both on expression of cell viability markers and the oxidative stress in differentiated HT22. (a–c) Western blotting analysis for Akt and B-cell lymphoma 2 (Bcl-2): RPM exposure significantly reduced Akt and Bcl-2 expressions, while treated cells showed similar or higher levels of these proteins compared with the normogravity regime. (d) Intracellular levels of reactive oxygen species (ROS) measurement (*n* = 15 from *N* = 5 experiments): RPM exposure promoted a significant increase in oxidative stress (****p<0.0001), which was completely counteracted by combined treatment; whereas treatment with Trolox or r-Irisin only partially reduced the oxidative stress induced by RPM exposure. Data were expressed as mean ± standard deviation and were compared by one-way ANOVA and Tukey’s multiple comparison test. (e–i) Immunofluorescence analysis for NADPH oxidase 4 (NOX4): Nuclei are stained with DAPI (blue) in HT22 cells, while NOX4 immunostaining is depicted in red (arrows) in HT22 cells. 40× images, scale bar represents 100 μm.

These results were confirmed by western blotting analysis with anti-Akt and anti-Bcl-2 antibodies (Fig 3A–3C). Particularly, densitometric analysis shown in Fig 3B shows a dramatic reduction in Akt expression in RPM-exposed cells compared with control cells (1.75 ± 0.07 *vs* 0.99 ± 0.03, ****p<0.0001). In contrast, Trolox treatment preserved the expression of this protein, the levels of which were like those of control cells (1.76 ± 0.05); while a significant increase in Akt expression was detected in r-Irisin-treated cells (1.82 ± 0.08, *p<0.05). Importantly, the highest expression of Akt was detected in the differentiated HT22 cells undergoing combined treatment, with significantly higher values than in control cells (2.17 ± 0.08, ****p<0.0001). In agreement, the Bcl-2 expression (Fig 3C) was significantly reduced after RPM exposure (0.24 ± 0.01, ****p<0.0001) compared to the normogravity condition (0.47 ± 0.03). In contrast, treatment with Trolox (0.49 ± 0.02, *p<0.05) or r-Irisin (0.48 ± 0.01) preserved the levels of this protein, with values like those of control cells. Interestingly, a significant increase in Bcl-2 expression was detected in cells exposed to combined treatment, as the values obtained by densitometric analysis were significantly higher (0.68 ± 0.04, ****p<0.0001) than in untreated cells maintained under normal conditions.

Finally, measurement of intracellular ROS levels (Fig 3D) showed a significant increase in oxidative stress in differentiated HT22 cells exposed to 48 h of RPM compared with those maintained under normogravity (100.0 ± 13.9 *vs* 177.4 ± 24.6, ****p<0.0001). Surprisingly, combined treatment with Trolox and r-Irisin completely counteracted this increase, returning intracellular ROS levels to values like control (105.6 ± 14.9). Treatment with Trolox or r-Irisin also reduced the oxidative stress induced by RPM exposure, albeit partially, maintaining intracellular ROS levels at significantly higher values than those found under normogravity (Trolox Treatment: 121.9 ± 14.9, **p<0.01; r-Irisin Treatment: 121.0 ± 22.3, *p<0.05).

In agreement, immunofluorescence analysis detected marked NOX4 expression in RPM-exposed cells respect to normogravity regime (Fig 3E and 3F). Noteworthy, cells treated with Trolox (Fig 3G) or r-Irisin (Fig 3H) were characterized by a reduced fluorescent signal; whereas NOX4 expression comparable to that of the control was observed in cells receiving combined treatment (Fig 3I).

### BDNF expression is preserved in treated cells exposed to RPM

The potential efficacy of treatment with Trolox, r-Irisin or both in counteracting cognitive decline induced by RPM exposure was evaluated by investigating the BDNF expression, a neurotrophin essential for neuronal development and survival, synaptic plasticity, and cognitive function, by immunocytochemistry and western blotting.

**Fig 4:**
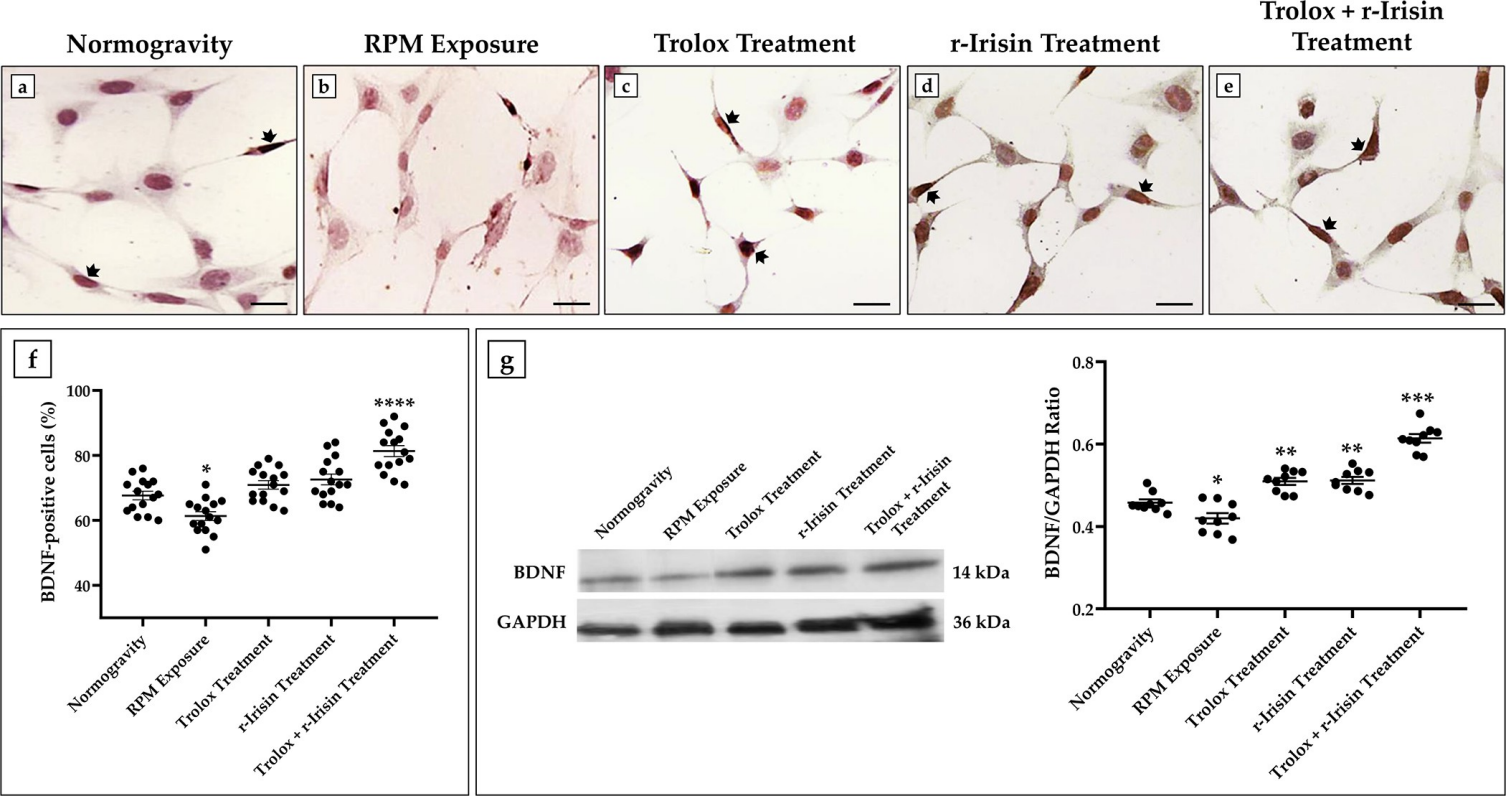
Effects of treatment with Trolox, r-Irisin or both on brain-derived neurotrophic factor (BDNF) expression in differentiated HT22 cells. (a–f) Immunocytochemical analysis: RPM exposure significantly reduced the percentage of BDNF-positive cells respect to normogravity regime (a,b), which was unchanged after treatment with Trolox (c) and r-Irisin (d). The highest BDNF expression was observed when cells were subjected to combined treatment (e). In each panel, arrows indicate the presence of BDNF-positive cells (*n* = 15 from *N* = 5 experiments). 40× images, scale bar represents 100 μm. (g) Western blotting analysis for BDNF: RPM exposure significantly reduced BDNF expressions, while treated cells showed higher levels of these proteins compared with the normogravity regime. Data were expressed as mean ± standard deviation and were compared by one-way ANOVA and Tukey’s multiple comparison test.

Immunocytochemical analysis revealed marked differences in BDNF expression depending on the experimental condition (Fig 4A–4F). First, a significant reduction in BDNF expression was observed in cells exposed to RPM compared with those maintained in normogravity (67.7 ± 5.2 *vs* 61.3 ± 5.2, *p<0.05). Fortunately, this effect was neutralized by Trolox or r-Irisin treatment, with BDNF expression values like those of cells in normogravity (70.9 ± 5.1 and 72.6 ± 6.47, respectively). Noteworthy, the higher expression of neurotrophin was observed in differentiated HT22 cells treated with Trolox and r-Irisin, since the percentage of cells positive for BDNF was significantly higher than control cells (81.3 ± 6.6, ****p<0.0001).

The immunocytochemistry results were confirmed by western blotting analysis, which showed a positive band at approximately 14 kDa, corresponding to the molecular weight of monomeric BDNF, in the protein extracts of all cell samples (Fig 4G). Specifically, BDNF expression, which was significantly reduced after RPM exposure (0.42 ± 0.04, *p<0.05), was preserved by Trolox (0.51 ± 0.03, **p<0.01) or r-Irisin treatment (0.52 ± 0.03, **p<0.01), with values significantly higher than those observed in untreated cells (0.46 ± 0.02). Interestingly, the highest BDNF expression levels were detected in the differentiated HT22 cells under combined treatment, as the values obtained from densitometric analysis were significantly higher than in the normogravity regime (0.61 ± 0.03, ***p<0.001).

## Discussion

Although there are numerous physiological changes induced by exposure to microgravity, the occurrence of neurological deficits is certainly one of the most worrisome [39]. The brain deficits observed in astronauts exposed to spaceflight are structural and functional, including reduced postural and locomotor control [40], upward displacement of the brain within the skull [41], disruption of structural white matter connectivity [42], increased fluid volume [43], as well as alterations in adaptive plasticity [44].

From a molecular perspective, oxidative stress has been suggested as an important culprit responsible for death in numerous cell types [45–47]. Indeed, our results clearly show that differentiated HT22 cells exposed to RPM undergo a dramatic increase in intracellular ROS and undergo marked apoptotic death, as shown by altered expression of Akt, Bcl-2 and NOX4. Interestingly, the biological effects of microgravity on nerve cells also include reduced expression of BDNF, an important marker of brain health, neurogenesis, and cognitive function, suggesting the possibility of extensive systemic alterations in the CNS induced by weightlessness [48,49].

Therefore, a potential countermeasure should include antioxidant strategies aimed at counteracting increased ROS production and preventing neuronal death. In this regard, Trolox has been reported to be a potent, fat-soluble, fast-acting free radical scavenger with more potent antioxidant efficacy than its original compound, vitamin E [50]. Morabito et al. have previously observed the efficacy of Trolox in the osteoblastic MC3T3-E1 cell line exposed to RPM in terms of intracellular ROS, intracellular calcium, and mitochondrial function [51]. Irisin, whose expression increases with exercise, has also been suggested to play a neuroprotective role by stimulating neurogenesis and increasing BDNF expression [52]. These two strategies, alone or in combination, could be valuable countermeasures to counteract neuronal depletion in microgravity and pave the way for further research aimed at preserving brain health under adverse conditions.

The Trolox dose-response curve establishment was instrumental in identifying the appropriate concentration to be administered. In agreement with Morabito et al. [51], the dose-response curve showed that the optimal administration concentration was 1 × 10^−4^ M and that cell viability was reduced by 50% at the concentration of 1 × 10^−3^ M. Notably, at the concentration of 1 × 10^−4^ M, Trolox strongly reduced intracellular ROS levels and NOX4 expression in differentiated HT22 cells, although these were still significantly higher than those in cells maintained in normogravity regime. In addition, Trolox treatment promoted a significant increase in the Akt and Bcl-2 expressions, resulting in a gain in the capacity for cell proliferation and survival. BDNF expression was also preserved by Trolox treatment, suggesting a role of this antioxidant in counteracting the cognitive decline induced by weightlessness.

Importantly, similar results have been obtained by treatment of differentiated HT22 cells with r-Irisin. Although a role for it in counteracting the neuronal alterations induced by weightlessness has not yet been demonstrated, several evidences have proposed irisin as a critical regulator of cognitive function [53], being able to inhibit oxidative stress [54], promote BDNF accumulation in hippocampal neurons [55,56], improve synaptic plasticity in mouse models of Alzheimer’s disease [57] and prevent neurodegeneration in mouse models of Parkinson’s disease [58]. Therefore, its therapeutic potential might not be limited to neurodegenerative diseases but also extend to preventing neuronal deficits that occur during spaceflight.

However, the efficacy of r-Irisin could be limited over time due to the short half-life of the protein [24]. Therefore, we hypothesised that a combined treatment with Trolox and r-Irisin may provide greater protection against damage induced by RPM exposure. Surprisingly, better effects were observed with the combination treatment, as intracellular ROS levels and NOX4 expression were restored to control values. Noteworthy, preservation of cell viability was associated with increased BDNF expression, with values above those observed under normogravity conditions.

To our knowledge, few studies aim to identify a potential strategy for counteracting neuronal death induced by unloading. Among these, Qu and colleagues in 2010 investigated the efficacy of flavonoids, substances with antioxidant power, in counteracting ROS increase in the neuroblastoma line SH-SY5Y, demonstrating a crucial role of oxidative stress in neuronal death induced by simulated microgravity [59]. Furthermore, Jiang et al. recently evaluated the efficacy of some steroid components of ginseng in preserving memory in a simulated microgravity model. Specifically, the authors administered diol-type ginseng saponin (Rb1) and triol-type ginseng saponin (Rg1) to rats subjected to limb unloading and observed a reduction in ROS in the prefrontal cortex, concomitant with a reduction in markers of apoptosis and an increase in BDNF expression compared to untreated rats [60]. This evidence confirms the responsibility of oxidative stress in microgravity-induced neuronal death and suggests the existence of a worrying alteration in the mechanisms underlying memory and learning processes.

Overall, the results obtained show that a combined treatment with Trolox and r-Irisin may be an effective solution in reducing oxidative stress and apoptotic death and preserving BDNF expression. However, further research should be conducted to determine whether this combination is also effective for longer periods of time or whether further administration is needed. In addition, the development of further countermeasures requires a more thorough elucidation of all biological mechanisms involved in weightlessness-induced neuronal death, including determining the molecular basis of the potential synergistic action between Trolox and r-Irisin.

## Conclusions

Among the many challenges to ensuring adequate safety during space travel, nerve and cognitive alterations are among the most important. The biological effects of microgravity on the nervous system include increased oxidative stress, apoptotic death of neurons and reduced BDNF expression. Identifying strategies to counteract these alterations should be the main concern of space biomedicine. In this context, a combined treatment with Trolox and r-Irisin could provide a viable defense strategy to safeguard the health of neurons. Although the combined treatment of Trolox and r-Irisin has shown positive results, further studies are needed to thoroughly evaluate the effectiveness of this combination and identify further countermeasures to ensure cognitive health during spaceflight.

### Limits of the study

The main limitation of this study is that the administration of substances with effects on hippocampal neurons could have significantly different effects in vivo. Indeed, the complexity of the nervous tissue, characterized by glial cells, blood vessels and supporting structures, could profoundly influence the absorption of the substances analyzed in vitro. Therefore, it should be emphasized that our results demonstrate the beneficial effect of r-Irisin and Trolox on the health of neurons in culture, without considering the complex network of the nervous system that could influence neuronal responses to treatment.

Furthermore, although RPM is a widely accepted tool for simulating weightlessness, it cannot reproduce the same microgravity conditions experienced by space crews. Indeed, such an instrument is an excellent solution for reproducing the biological effects of microgravity, but the evidence should be corroborated by experiments conducted outside Earth orbit in real microgravity conditions.

## Supporting information

S1 Fig Brightfield images of differentiated HT22 cells before and after exposure to RPM. Untreated HT22 cells before (a) and after exposure to RPM (b). HT22 cells before (c) and after exposure to RPM (d) treated with Trolox. HT22 cells before (e) and after exposure to RPM (f) treated with r-Irisin. HT22 cells before (g) and after exposure to RPM (h) treated with Trolox and r-Irisin. 40× images, scale bar represents 100 μm.
(TIF)

S2 Fig Immunocytochemistry and original western blotting images. (a) Negative control of HT22 cells for brain-derived neurotrophic factor (BDNF) by immunocytochemistry. 40× images, scale bar represents 100 μm. (b–e) Original western blotting images: (b) The band shown corresponds to Akt, with a molecular weight of 60 kDa; (c) The band shown corresponds to B-cell lymphoma 2 (Bcl-2), with a molecular weight of 26 kDa; (d) The band shown corresponds to BDNF, with a molecular weight of 14 kDa; (e) The band shown corresponds to GAPDH, with a molecular weight of 36 kDa.
(TIF)

S1 Table Cell viability data by MTS assay. (DOCX)

S2 Table Akt/GAPDH ratio data. (DOCX)

S3 Table Bcl-2/GAPDH ratio. (DOCX)

S4 Table Intracellular ROS level data. (DOCX)

S5 Table BDNF immunocytochemistry data. (DOCX)

S6 Table BDNF/GAPDH ratio data. (DOCX)
