# Supplementary figures and images for: Trolox and recombinant Irisin as a potential strategy to prevent neuronal damage induced by random positioning machine exposure in differentiated HT22 cells
Source: PLoS One. 2024 Mar 21;19(3):e0300888. doi: 10.1371/journal.pone.0300888 (PMC10956770; doi:10.1371/journal.pone.0300888)

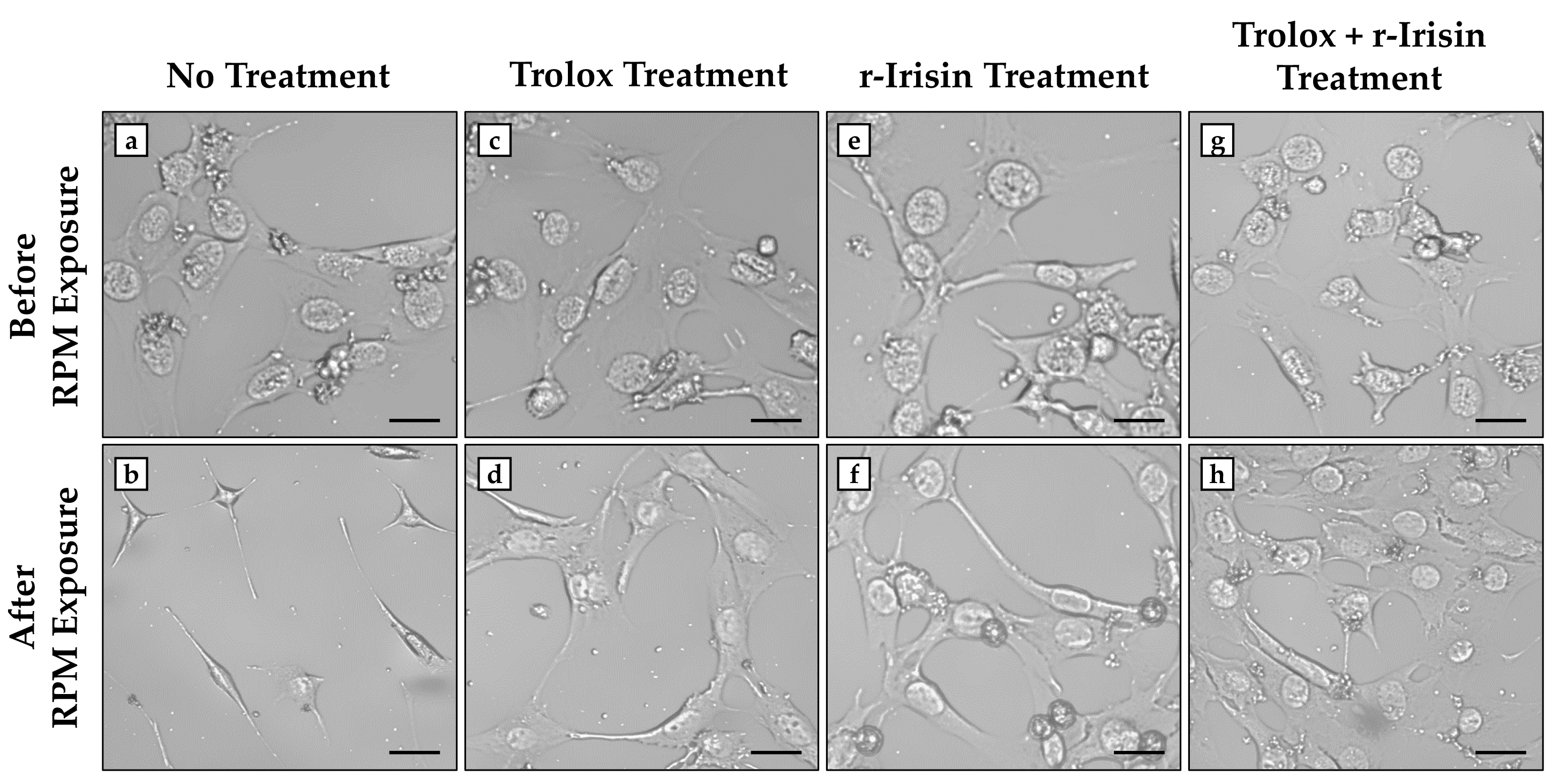

Supplement: S1 Fig — Untreated HT22 cells before (a) and after exposure to RPM (b). HT22 cells before (c) and after exposure to RPM (d) treated with Trolox. HT22 cells before (e) and after exposure to RPM (f) treated with r-Irisin. HT22 cells before (g) and after exposure to RPM (h) treated with Trolox and r-Irisin. 40× images, scale bar represents 100 μm. (TIF) [file pone.0300888.s001.tif]

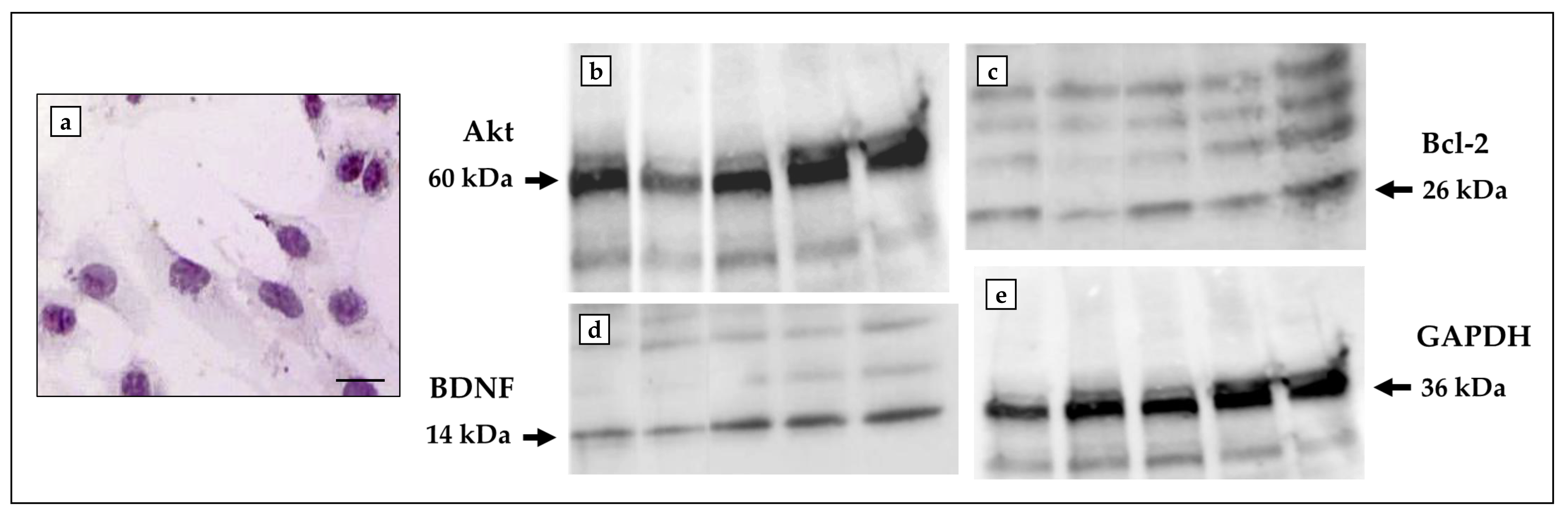

Supplement: S2 Fig — (a) Negative control of HT22 cells for brain-derived neurotrophic factor (BDNF) by immunocytochemistry. 40× images, scale bar represents 100 μm. (b–e) Original western blotting images: (b) The band shown corresponds to Akt, with a molecular weight of 60 kDa; (c) The band shown corresponds to B-cell lymphoma 2 (Bcl-2), with a molecular weight of 26 kDa; (d) The band shown corresponds to BDNF, with a molecular weight of 14 kDa; (e) The band shown corresponds to GAPDH, with a molecular weight of 36 kDa. (TIF) [file pone.0300888.s002.tif]
