# Supplementary material for: Trolox and recombinant Irisin as a potential strategy to prevent neuronal damage induced by random positioning machine exposure in differentiated HT22 cells
Source: PLoS One. 2024 Mar 21;19(3):e0300888. doi: 10.1371/journal.pone.0300888 (PMC10956770; doi:10.1371/journal.pone.0300888)
Supplement: S1 Table — (DOCX) [file pone.0300888.s003.docx]

**Table S1. Cell viability data by MTS assay.**

|  | **Normogravity** | **RPM Exposure** | **Trolox Treatment** | **r-Irisin Treatment** | **Trolox +**  **r-Irisin Treatment** |
| --- | --- | --- | --- | --- | --- |
|  | \| 98,45628925 \| \| --- \| \| 101,3448183 \| \| 96,13464983 \| \| 100,6380188 \| \| 108,7188521 \| \| 90,8219675 \| \| 97,19376999 \| \| 100,3734907 \| \| 106,3181436 \| \| 109,7188521 \| \| 98,8219675 \| \| 92,19376999 \| \| 89,37349066 \| \| 99,31814361 \| \| 110,8219675 \| | \| 88,946646 \| \| --- \| \| 53,60852876 \| \| 71,53594753 \| \| 77,98815398 \| \| 83,69002655 \| \| 75,81704685 \| \| 60,20519836 \| \| 65,63397921 \| \| 75,10502961 \| \| 68,946646 \| \| 73,60852876 \| \| 61,53594753 \| \| 57,98815398 \| \| 73,69002655 \| \| 79,81704685 \| | \| 85,27557747 \| \| --- \| \| 91,82975301 \| \| 79,70205529 \| \| 84,59235909 \| \| 76,31405804 \| \| 84,24106901 \| \| 85,04155827 \| \| 94,46058838 \| \| 98,26485015 \| \| 95,27557747 \| \| 96,82975301 \| \| 79,70205529 \| \| 81,59235909 \| \| 86,31405804 \| \| 78,82975301 \| | \| 84,18901997 \| \| --- \| \| 93,25004793 \| \| 91,81639037 \| \| 102,2591756 \| \| 85,56333658 \| \| 78,12043053 \| \| 99,17526643 \| \| 101,682001 \| \| 95,22558647 \| \| 84,25917563 \| \| 80,56333658 \| \| 91,12043053 \| \| 103,1752664 \| \| 104,682001 \| \| 82,22558647 \| | \| 122,6269587 \| \| --- \| \| 118,459643 \| \| 127,7388336 \| \| 125,9252575 \| \| 106,3124785 \| \| 123,0590176 \| \| 118,1487563 \| \| 110,2956487 \| \| 120,7698563 \| \| 110,3124785 \| \| 129,0590176 \| \| 118,1487563 \| \| 111,2956487 \| \| 119,7698563 \| \| 128,0590176 \| |
| **Media** | 100,0165461 | 71,20779377 | 86,55102831 | 91,8204701 | 119,3320817 |
| **SD** | 6,627901948 | 9,907813689 | 7,098099741 | 8,983430011 | 7,134361515 |
