# Supplementary material for: Trolox and recombinant Irisin as a potential strategy to prevent neuronal damage induced by random positioning machine exposure in differentiated HT22 cells
Source: PLoS One. 2024 Mar 21;19(3):e0300888. doi: 10.1371/journal.pone.0300888 (PMC10956770; doi:10.1371/journal.pone.0300888)
Supplement: S2 Table — (DOCX) [file pone.0300888.s004.docx]

**Table S2. Akt/GAPDH ratio data.**

|  | **Normogravity** | **RPM Exposure** | **Trolox Treatment** | **r-Irisin Treatment** | **Trolox +**  **r-Irisin Treatment** |
| --- | --- | --- | --- | --- | --- |
|  | 1,757463727  1,819309483  1,713829784  1,669460083  1,748475786  1,67935031  1,757000419  1,896409611  1,679179016 | 1,022198549  0,986394285  0,995840131  1,033560741  0,991543142  1,011459955  1,039164527  0,940399927  0,947440533 | 1,842967455  1,727550253  1,722782946  1,77519096  1,737895794  1,700771821  1,787874081  1,785132331  1,801492527 | 1,819030779  1,94700356  1,840542073  1,853061083  1,729364423  1,895568845  1,826367773  1,727453882  1,746567649 | 2,078499779  2,18498327  2,233737071  2,064852292  2,215137686  2,232304241  2,041935305  2,206324453  2,256079676 |
| **Media** | 1,746719802 | 0,996444643 | 1,764628685 | 1,820551118 | 2,168205975 |
| **SD** | 0,074205568 | 0,03492694 | 0,045396087 | 0,075470377 | 0,082715815 |
