# Supplementary material for: Trolox and recombinant Irisin as a potential strategy to prevent neuronal damage induced by random positioning machine exposure in differentiated HT22 cells
Source: PLoS One. 2024 Mar 21;19(3):e0300888. doi: 10.1371/journal.pone.0300888 (PMC10956770; doi:10.1371/journal.pone.0300888)
Supplement: S3 Table — (DOCX) [file pone.0300888.s005.docx]

**Table S3. Bcl-2/GAPDH ratio.**

|  | **Normogravity** | **RPM Exposure** | **Trolox Treatment** | **r-Irisin Treatment** | **Trolox +**  **r-Irisin Treatment** |
| --- | --- | --- | --- | --- | --- |
|  | 0,488243416  0,478369255  0,422596337  0,452897878  0,471304234  0,427707014  0,493544988  0,491983651  0,481222516 | 0,258993867  0,23700407  0,237657476  0,253345935  0,237462138  0,239000551  0,245349914  0,223474337  0,2364775 | 0,497411939  0,501526391  0,497409186  0,511216909  0,465595214  0,493504909  0,515933175  0,474367552  0,50872708 | 0,488056194  0,498523207  0,48717148  0,476154498  0,474716885  0,496368117  0,466397837  0,472303721  0,465061556 | 0,659580356  0,674173524  0,640799658  0,678024823  0,744642302  0,708709344  0,675848018  0,683892041  0,624449212 |
| **Media** | 0,467541032 | 0,240973976 | 0,496188039 | 0,480528166 | 0,67667992 |
| **SD** | 0,027023202 | 0,010406567 | 0,016672229 | 0,012424542 | 0,035379023 |
