# Supplementary material for: Trolox and recombinant Irisin as a potential strategy to prevent neuronal damage induced by random positioning machine exposure in differentiated HT22 cells
Source: PLoS One. 2024 Mar 21;19(3):e0300888. doi: 10.1371/journal.pone.0300888 (PMC10956770; doi:10.1371/journal.pone.0300888)
Supplement: S4 Table — (DOCX) [file pone.0300888.s006.docx]

**Table S4. Intracellular ROS level data.**

|  | **Normogravity** | **RPM Exposure** | **Trolox Treatment** | **r-Irisin Treatment** | **Trolox +**  **r-Irisin Treatment** |
| --- | --- | --- | --- | --- | --- |
|  | \| 106,4315732 \| \| --- \| \| 98,83975882 \| \| 90,69979901 \| \| 93,22126804 \| \| 114,4344966 \| \| 100,8404897 \| \| 99,90864243 \| \| 125,0137036 \| \| 75,54357756 \| \| 80,36725745 \| \| 101,4708569 \| \| 99,2508679 \| \| 94,50940983 \| \| 125,2329618 \| \| 94,23533711 \| | \| 162,2419149 \| \| --- \| \| 188,4980815 \| \| 208,4505756 \| \| 137,4931482 \| \| 161,9952494 \| \| 216,8372008 \| \| 196,9075461 \| \| 182,7653938 \| \| 140,9693039 \| \| 162,9819112 \| \| 193,9795359 \| \| 158,3774895 \| \| 207,5735428 \| \| 161,7759912 \| \| 180,2758999 \| | \| 119,125708 \| \| --- \| \| 110,355381 \| \| 107,6694683 \| \| 130,9656496 \| \| 124,3056824 \| \| 143,1618856 \| \| 114,8227663 \| \| 140,7500457 \| \| 144,6418783 \| \| 96,6791522 \| \| 106,627992 \| \| 128,0604787 \| \| 124,8264206 \| \| 104,2709666 \| \| 132,6649004 \| | \| 161,2369815 \| \| --- \| \| 120,4549607 \| \| 135,693404 \| \| 131,6919423 \| \| 119,9068153 \| \| 123,0860588 \| \| 137,3926549 \| \| 152,0555454 \| \| 106,2031792 \| \| 86,66179426 \| \| 87,40179061 \| \| 112,8083318 \| \| 96,93952129 \| \| 104,6957793 \| \| 139,2289421 \| | \| 115,461356 \| \| --- \| \| 76,8171021 \| \| 92,7133199 \| \| 108,554723 \| \| 115,790243 \| \| 94,4673853 \| \| 97,2629271 \| \| 125,602046 \| \| 80,6815275 \| \| 110,034716 \| \| 114,529508 \| \| 107,07473 \| \| 127,136854 \| \| 102,415494 \| \| 115,762836 \| |
| **Media** | 100 | 177,4081856 | 121,9285584 | 121,0305134 | 105,620318 |
| **SD** | 13,87772405 | 24,56216157 | 14,99672114 | 22,29550599 | 14,8747758 |
