# Supplementary material for: Trolox and recombinant Irisin as a potential strategy to prevent neuronal damage induced by random positioning machine exposure in differentiated HT22 cells
Source: PLoS One. 2024 Mar 21;19(3):e0300888. doi: 10.1371/journal.pone.0300888 (PMC10956770; doi:10.1371/journal.pone.0300888)
Supplement: S5 Table — (DOCX) [file pone.0300888.s007.docx]

**Table S5. BDNF immunocytochemistry data.**

|  | **Normogravity** | **RPM Exposure** | **Trolox Treatment** | **r-Irisin Treatment** | **Trolox +**  **r-Irisin Treatment** |
| --- | --- | --- | --- | --- | --- |
|  | \| 75 \| \| --- \| \| 67 \| \| 68 \| \| 61 \| \| 72 \| \| 63 \| \| 71 \| \| 64 \| \| 72 \| \| 71 \| \| 76 \| \| 61 \| \| 69 \| \| 60 \| \| 65 \| | \| 64 \| \| --- \| \| 65 \| \| 57 \| \| 55 \| \| 59 \| \| 66 \| \| 51 \| \| 63 \| \| 61 \| \| 57 \| \| 71 \| \| 59 \| \| 65 \| \| 67 \| \| 60 \| | \| 74 \| \| --- \| \| 77 \| \| 69 \| \| 75 \| \| 73 \| \| 71 \| \| 68 \| \| 68 \| \| 64 \| \| 74 \| \| 79 \| \| 66 \| \| 77 \| \| 66 \| \| 63 \| | \| 75 \| \| --- \| \| 80 \| \| 69 \| \| 72 \| \| 65 \| \| 69 \| \| 78 \| \| 84 \| \| 71 \| \| 64 \| \| 68 \| \| 75 \| \| 83 \| \| 65 \| \| 71 \| | \| 90 \| \| --- \| \| 89 \| \| 87 \| \| 72 \| \| 78 \| \| 85 \| \| 79 \| \| 92 \| \| 74 \| \| 81 \| \| 77 \| \| 84 \| \| 71 \| \| 77 \| \| 84 \| |
| **Media** | 67,66666667 | 61,33333333 | 70,93333 | 72,6 | 81,3333333 |
| **SD** | 5,177791403 | 5,219012861 | 5,063407 | 6,412042 | 6,59725773 |
