# Supplementary material for: Trolox and recombinant Irisin as a potential strategy to prevent neuronal damage induced by random positioning machine exposure in differentiated HT22 cells
Source: PLoS One. 2024 Mar 21;19(3):e0300888. doi: 10.1371/journal.pone.0300888 (PMC10956770; doi:10.1371/journal.pone.0300888)
Supplement: S6 Table — (DOCX) [file pone.0300888.s008.docx]

**Table S6. BDNF/GAPDH ratio data.**

|  | **Normogravity** | **RPM Exposure** | **Trolox Treatment** | **r-Irisin Treatment** | **Trolox +**  **r-Irisin Treatment** |
| --- | --- | --- | --- | --- | --- |
|  | 0,442855478  0,485982034  0,429987213  0,456912831  0,505539304  0,446079627  0,453658898  0,45109499  0,449527113 | 0,454112922  0,423788617  0,46852174  0,380864439  0,368007344  0,411501417  0,469911206  0,41443188  0,386045408 | 0,533993865  0,473865833  0,540388994  0,521244463  0,532071405  0,473155659  0,486162024  0,50900829  0,514908044 | 0,485764621  0,490133834  0,507663336  0,527138445  0,47616427  0,552591345  0,500999535  0,534492854  0,531233136 | 0,628579691  0,621355304  0,673920028  0,569318022  0,633294223  0,573229928  0,612020061  0,609016866  0,604675229 |
| **Media** | 0,457959721 | 0,419687219 | 0,509422064 | 0,511797931 | 0,613934372 |
| **SD** | 0,023287951 | 0,037936831 | 0,025899518 | 0,025842661 | 0,031630063 |
